# Automatic engagement of limbic and prefrontal networks in response to food images reflects distinct information about food hedonics and inhibitory control

**DOI:** 10.1038/s42003-025-07704-w

**Published:** 2025-02-20

**Authors:** Jason A. Avery, Madeline Carrington, John E. Ingeholm, Valerie Darcey, W. Kyle Simmons, Kevin D. Hall, Alex Martin

**Affiliations:** 1 Laboratory of Brain and Cognition, National Institute of Mental Health, Bethesda, MD USA; 2 Integrative Physiology Section, National Institute of Diabetes & Digestive & Kidney Diseases, Bethesda, MD USA; 3 Department of Pharmacology and Physiology, Oklahoma State University Center for Health Sciences, Tulsa, OK USA

**Keywords:** Feeding behaviour, Cognitive control

## Abstract

Adaptive regulation of food consumption involves both identifying food as well as evaluating whether it should be eaten, a process that requires assessing relevant properties such as healthfulness and hedonic value. In order to identify how these fine-grained food properties are represented in the brain, we analyzed functional Magnetic Resonance Imaging data from 43 participants who viewed images of 36 different foods. A data-driven clustering approach based on Representational Similarity Analysis partitioned food-responsive brain regions into two sub-networks based on their multivariate response to food pictures: a Prefrontal network composed of fronto-parietal brain regions and a Limbic network composed of cortico-limbic and sub-cortical brain regions. Further analysis, using similarity judgments of those foods from a large online sample, revealed that the Prefrontal network predominantly represented information related to food healthfulness or processing, the key factor underlying food similarity. In another imaging task, we found that responses in the Prefrontal network were strongly influenced by judgments of food-related self-control, while the Limbic network responses were more affected by hedonic food judgments. These results suggest that, upon viewing food images, behaviorally relevant information is automatically retrieved from distinct brain networks that act as opponent processes in guiding food consumption.

## Abstract (web summary)

Representational similarity analysis reveals distinct networks of prefrontal and limbic brain regions that automatically encode healthfulness and hedonic value, guiding food consumption.

## Introduction

Brain responses to food stimuli have been a topic of interest for nearly 20 years in the field of neuroimaging research. Meta-analyses of neuroimaging studies involving food picture stimuli have shown that there are a few regions that are consistently responsive to food images, compared to scenes or non-food objects, including the orbitofrontal cortex, amygdala, and insular cortex^1–3^. Some recent studies have also suggested that ventral visual regions exhibit a degree of selectivity for food images^4–6^.

Despite this, there is still much to be learned about how the brain responds to food. Studies that have examined the neural response to food-related stimuli (most commonly images of foods displayed to subjects during fMRI scanning) in healthy populations have come in two general varieties. Studies of object representation that use foods as one of several different object categories (e.g., faces, tools, scenes) often group all foods together as a single category^7–9^, an approach which usually does not account for the broad variation in properties present across different foods. In contrast, behavioral and clinical studies focus more on the nutritional composition of depicted foods, such as fat, sugar, or caloric content^10,11^. While this approach does focus on factors which are more clinically relevant, it overlooks other relevant food properties that can also drive food-related decision-making (such as taste, the appearance of food, or if foods “go together”^12^). This approach can also lead to the generation of food groups which are somewhat counterintuitive. For example, an approach that grouped foods together based on sugar and fat content might group fruit salad and marshmallows together within the same sub-category^13^, though these foods are wildly dissimilar across many relevant dimensions relevant to health (e.g., degree of food processing^14^).

The univariate-contrast approach typically employed for food-related neuroimaging studies also has some limitations. In general, univariate analyses enable researchers to identify regions in which the activation for a particular object category, such as food, is greater than activation for other objects. However, they do not indicate what type of information about food is being represented, nor whether that same information is shared across multiple food-responsive regions. In contrast, multivariate techniques such as Representational Similarity Analysis (RSA^15,16^) can be used to identify the presence of property-specific information within a brain region, as well as compare the representation of information across regions, thus affording increased inferential power over standard univariate methods.

In the present study, we sought to examine the representation of food-property information in the brain using complementary data-driven behavioral and neuroimaging analysis approaches. We employed data from an online behavioral study^17^ which used an odd-one-out triplet task^18,19^ to obtain similarity judgments of a set of food images used in previous neuroimaging studies^20–23^, images which had been previously selected by experimenters according to the nutritional content of the depicted foods. Analysis of the online task data revealed naturalistic food categories and dimensions that underlie the similarity judgments of those foods. We subsequently applied the results of these analyses to the analysis of neuroimaging data from a group of subjects who viewed images of these foods during fMRI scanning. We used multivariate RSA to examine where this information is represented, within brain regions preferentially responsive to foods (vs. non-food objects).

## Results

### Food pictures fMRI task

**Fig. 1:**
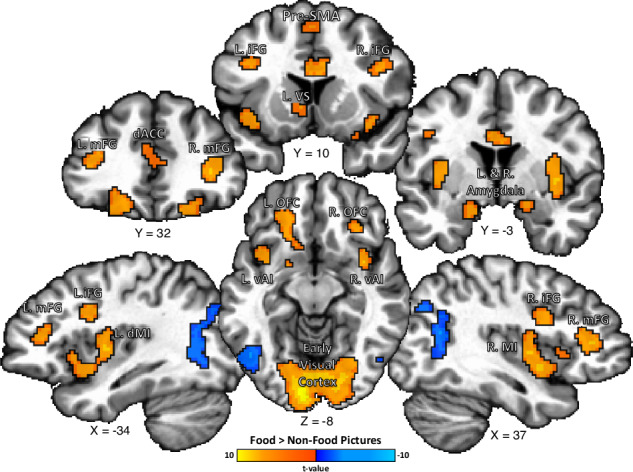
Hemodynamic Response to Food vs. Non-Food Images. A widespread number of brain regions are activated by images of foods, including regions involved in processing the sensory and affective components of food, such as the bilateral mid-insula (MI), orbitofrontal cortex (OFC), amygdala, and ventral striatum (VS), as well as fronto-parietal regions such as the bilateral inferior frontal gyrus (iFG) and medial frontal gyrus (mFG) and Pre-Supplemental Motor Area (Pre-SMA) involved in response inhibitional and behavioral control. vAI – ventral anterior insula, ACC – anterior cingulate cortex.

Forty-three healthy participants from the greater Washington, DC metropolitan area completed fMRI scans at the National Institutes of Health Clinical Center in Bethesda, MD. Participants were kept on a controlled and standardized eucaloric diet for at least 72 h prior to fMRI scanning (see “Methods” section for more details). The participants completed our Food Pictures task during functional Magnetic Resonance Imaging (fMRI), a task in which they viewed a broad selection of foods and non-food objects^20–23^. The 36 types of food presented in this task included foods high and low in both fat and sugar content, including appetizing foods such as French fries and donuts, as well as healthy food options such as fruits and vegetables. We performed a univariate fMRI analysis of the neuroimaging data from this task and identified several brain regions exhibiting a significantly greater hemodynamic response to food images than non-food images (Fig. 1). Those include classic ventral limbic and pre-limbic brain regions, such as bilateral regions of dorsal mid-insula and ventral anterior insula, bilateral regions of lateral orbitofrontal cortex (BA 11 m), bilateral regions of the amygdala, a region of the left ventral striatum bordering the nucleus accumbens, and a region of the dorsal anterior cingulate cortex (Fig. 1, Table S1). A food-specific response was also observed in dorsal and prefrontal regions of the brain, including regions such as the bilateral medial frontal gyrus and medial frontal gyrus, the right intraparietal sulcus, the right pre-central gyrus, and the pre-supplementary motor area. Food pictures also led to greater widespread activation of the early visual cortex extending into ventral occipitotemporal cortices, bilaterally, compared to non-food objects.

In contrast, a greater response to non-food objects was observed in bilateral regions of the lateral posterior temporal and parietal cortex (not listed), the right cuneus, and the left lingual gyrus (Fig. 1, Table S1).

### Identification of distinct food networks using RSA-based clustering of food-responsive regions

**Fig. 2:**
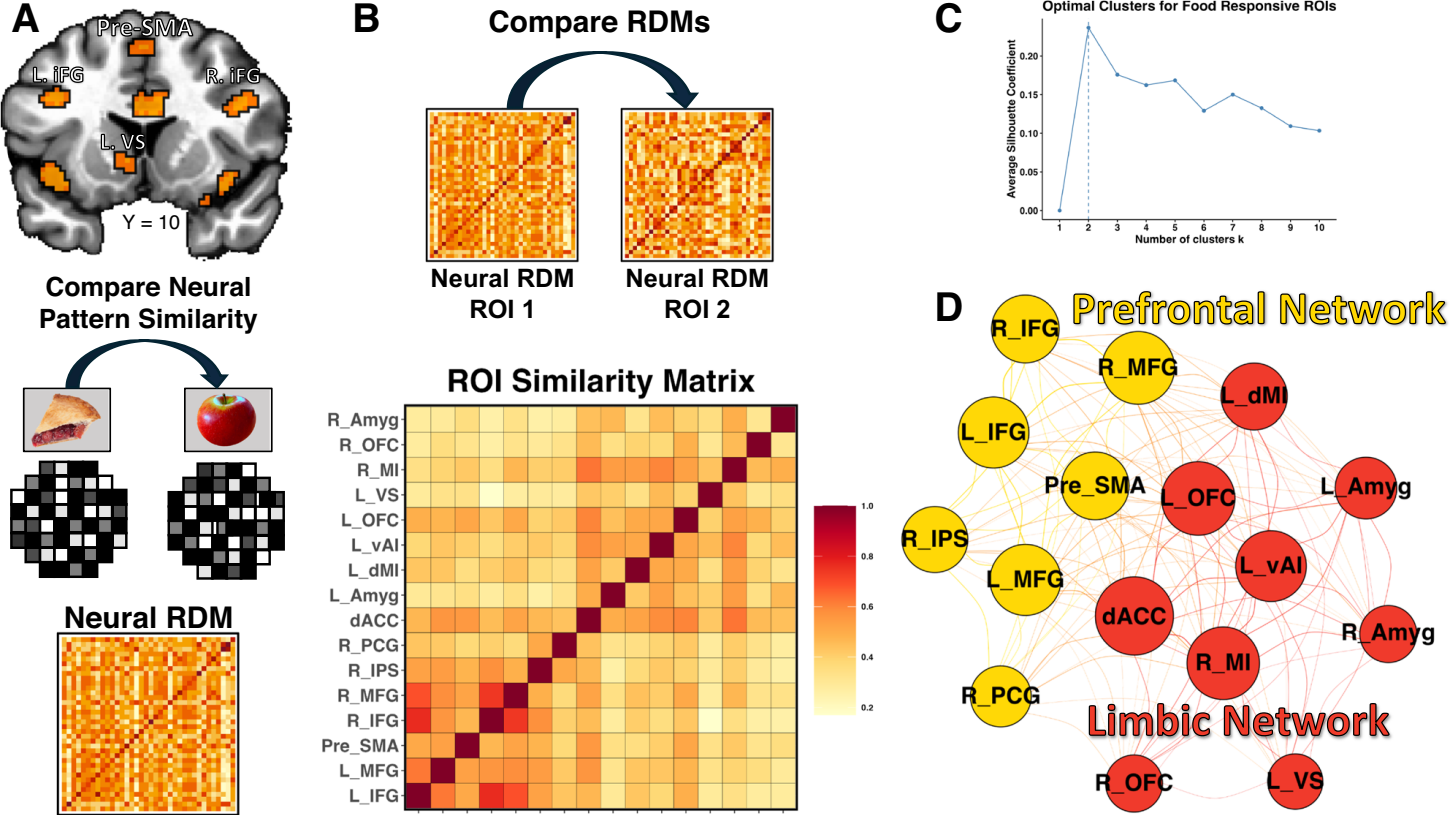
RSA-based clustering identifies separate prefrontal and limbic food networks. We applied a network clustering approach based on Representational Similarity Analysis (RSA^15^) to examine the representation of food images within food-responsive regions of the brain (**A**), (see Fig. 1). **A** We extracted and compared the multivariate food representations of each food from our food-responsive regions-of-interest (ROIs) to generate separate neural Representational Dissimilarity Matrices, **B** which we then compared across each pair of ROIs, resulting in a second-level ROI similarity matrix reflecting the similarity of representational profiles across our food-responsive regions. **C**, **D** We applied a network clustering algorithm to this matrix, which identified an optimal number of 2 clusters and partitioned the ROIs into two networks exhibiting categorically different representational profiles: a Prefrontal network composed of dorsal brain regions in prefrontal and parietal cortices, and a Limbic network composed of ventral cortico-limbic and sub-cortical brain regions. NB: Node sizes in this graph (**D**) are proportional to the average similarity of each node to every other node. The coloring of edge lines between nodes indicates whether those edges are within or between networks, and their thickness indicates the edge strength (i.e., similarity). The position of nodes within the graph indicates relative centrality within the whole node network. For copyright reasons, original images have been replaced with visually similar images from the public domain. Photos from Wikimedia.com. See the “Data availability” section for the location of original images.

We next ran a series of multivariate analyses to compare the representation of food-related information within those food-responsive brain regions. To accomplish this, we used a network clustering procedure, based on Representational Similarity Analysis (RSA), of the data from within those regions of interest (ROI). In general, this technique can be used to compare the responses to different stimuli across multiple behavioral and imaging modalities by representing those responses within a similarity space, which serves as a common reference frame^15^. As such, this method allowed us to examine the similarity of the neural response to food images across multiple food-responsive ROIs. Within each of the ROIs exhibiting a strong univariate response to food pictures (Figs. 1 and 2A), we extracted a neural Representational Dissimilarity Matrix (RDM), a matrix in which we compared the multivariate responses to each of the 36 foods presented in our task. We then compared the group average neural RDMs for each of these ROIs to create a new similarity matrix reflecting the similarity of each ROI representation of food-related information (Fig. 2B). We applied the k-means clustering algorithm to the resulting ROI similarity matrix (see “Methods” for details), and used a silhouette plot to identify the optimal clustering solutions (Fig. 2C), which split the food-responsive regions into two networks: (1) a “Prefrontal” network, composed of the dorsal food regions such as the medial and inferior frontal gyrus and the Pre-Supplementary Motor Area, and (2) the “Limbic” network, composed of regions such as the insula, orbitofrontal cortex, amygdala, and striatal regions (Fig. 2D).

### Behavioral RSA within food networks using online food similarity data

**Fig. 3:**
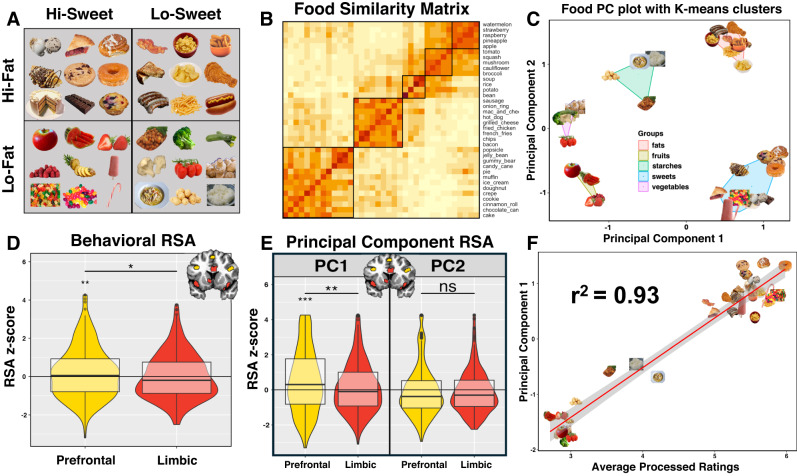
Information about food healthfulness is preferentially represented in the prefrontal food network. **A** An online study (Carrington et al., 2023) examined behavioral ratings of the similarity of the 36 foods, which were also presented to subjects within our neuroimaging task. Those foods were originally selected by researchers according to their relative fat and sugar content. **B** Analysis of the online ratings generated a food similarity matrix. **C** Clustering of the food similarity data along their Principal Component (PC) dimensions revealed five emergent food categories. **D** We performed an RSA where we compared the behavioral food similarity matrix to the neural RDMs, generated from the regions of our two food sub-networks (Fig. 2). Error bars represent the standard error of the mean. Boxplot lines signify distribution medians. Boxplot bottoms are distribution lower quartiles, and tops are upper quartiles. The RSA identified that the representation of food similarity information from the behavioral task was significantly greater in the Prefrontal network of brain regions. **E** This similarity between behavioral and neural data was due primarily to similarity along the first Principal Component dimension, (**F**) which was most related to ratings of how processed and unhealthy those foods were^17^. For copyright reasons, all original images have been replaced with visually similar images from the public domain. Photos from Pixabay.com and Wikimedia.com. See the “Data availability” section for the location of the original images.

For the following analyses, we employed the results of our online behavioral study^17^. This behavioral study involved an odd-one-out triplet task^18,19^, in which 487 online participants made similarity judgments of the 36 foods within our Food Pictures task (Fig. 3A, see “Methods” section for more details). The data from this task were used to create a pairwise food similarity matrix (Fig. 3B), which was then analyzed using Principal Components Analysis (PCA) and K-means clustering to identify naturalistic food categories that emerged within this food similarity space (‘fats’, ‘sweets’, ‘starches’, ‘fruits’, and ‘vegetables’; Fig. 3C).

When applying these behavioral results to the neuroimaging data, the first question we sought to answer was: in which of our two networks of food-responsive brain regions is this information about food similarity represented? To answer this question, we examined the relationship between a food RDM created from the online data and neural RDMs generated from our food-responsive ROIs (see “Methods” section for details) and then compared the resulting z-scored correlation values across our two food networks. We identified that this relationship was greatest in the Prefrontal food regions (*t*(14) = 2.16, *p* = 0.031; Table S2, Fig. 3D), suggesting that the relevant information about food similarity was best represented across this network of brain regions.

We conducted a subsequent RSA to identify which factors from the food similarity matrix were most strongly related to the neural similarity data. This RSA used separate RDMs created from the 1st two principal components of the food similarity matrix (Fig. 3C; see “Methods” section: Online Behavioral Study). The neural RDMs in the Prefrontal food network were significantly correlated to the 1st Principal Component RDM (Prefrontal: *t*(42) = 4.8, *p* < 0.001, Limbic: *t*(42) = 1.7, *p* = 0.088; Table S2, Fig. 3E). As with the overall similarity matrix, the relationship between neural similarity and behavioral similarity was greatest in the Prefrontal network of regions (*t*(14) = 3.03; *p* = 0.002). This first principal component, which reflected nearly half of the variability in these online similarity judgments, was shown to be nearly perfectly correlated with another group of participants’ ratings of how processed these foods were and their relative healthfulness^17^ (Fig. 3F). Notably, this relationship was still significant, even after regressing out the perceived and actual fat content of the foods. In contrast, neither sub-network showed a significant relationship with the 2nd Principal Component RDM (Prefrontal: *t*(42) = −1.17, *p* = 0.24, Limbic: *t*(42) = −1.66, *p* = 0.10; Table S2, Fig. 3E).

### Searchlight RSA results

**Fig. 4:**
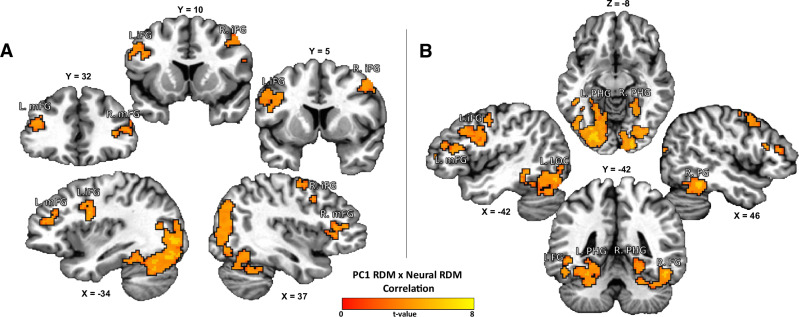
Searchlight RSA identifies prefrontal and ventral visual brain regions representing information related to food artificiality and healthfulness. We performed a multivariate searchlight RSA, comparing the neural dissimilarity to pictures of foods (Neural RDM) to the similarity of those foods along a dimension related to how processed, artificial, or (un)healthful those foods were (PC1 RDM; see Fig. 3C, F). **A** This multivariate analysis revealed multiple brain regions reflecting significant representational similarity between neural and model RDMs, including regions such as the bilateral medial frontal gyrus (mFG) and inferior frontal gyrus (iFG), regions which overlap with Prefrontal Food Network regions (see Fig. 2). **B** In the same analysis we also identified multiple regions in the ventral occipitotemporal cortex, such as the bilateral fusiform gyrus (FG), lateral occipital cortex (LOC), and (PHG) parahippocampal gyrus, regions that are typically associated with processing images of objects and visual scenes.

We next performed a multivariate searchlight RSA to verify the specificity of our ROI analysis results and identify other brain regions where the neural similarity of foods was significantly related to their similarity along behaviorally relevant property dimensions (the first two Principal Components from the behavioral food similarity data; Fig. 3B). As in our ROI analyses, we identified a significant relationship between neural similarity and similarity along the 1st Principal Component dimension in regions of the inferior frontal and middle frontal gyrus (Fig. 4A; Table S3). We also identified multiple regions within the occipital and ventral-temporal cortex where neural similarity was significantly correlated with the relative unhealthiness or artificiality of these foods (the 1st Principal Component), including regions involved in the visual processing of objects and scenes, such as the bilateral fusiform gyrus, lateral occipital cortex, and bilateral parahippocampal gyrus, respectively (Fig. 4B; Table S3). As in our ROI analyses above, we did not observe any brain regions exhibiting a significant positive relationship with the second principal component dimension, after correction for multiple comparisons.

### Food pleasantness and self-control (PSC) task

The results of our first neuroimaging experiment suggest that food-responsive regions of the brain cluster into two sub-networks with distinct representational profiles that reflect distinct types of food-relevant information. Our RSA results suggested that the Prefrontal network represented information related to the degree of food processing and healthfulness and did so to a significantly greater degree than the Limbic network. Given the association between the regions of this network and cognitive control, we theorized that this network would be involved in the behavioral regulation of food consumption. Based on prior studies of the Limbic network regions, we theorized that it was involved more in the appetitive response to food stimuli.

**Fig. 5:**
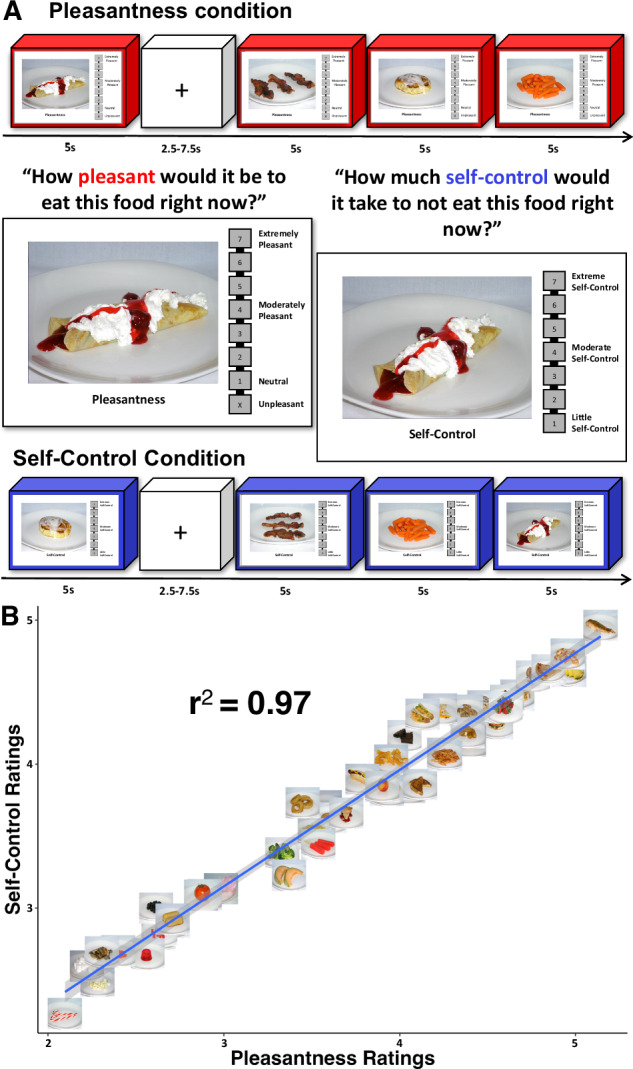
Food pleasantness and self-control task. **A** Participants performed another fMRI task in which they viewed a variety of foods that varied in nutritional content. During separate task blocks, they rated either the expected pleasantness of eating those foods or the degree of self-control required to NOT eat those foods. **B** Though the contextual framing of the conditions varied, participants’ average ratings across the pleasantness and self-control conditions were nearly perfectly correlated. All food images depicted here are original images prepared and photographed for this fMRI task. See the “Experimental design” section for more details.

In order to test these possibilities, we examined the data from a second functional neuroimaging task performed by the subjects in this study, in which they viewed another set of food pictures during fMRI scanning. During different blocks of the PSC task, they rated either the expected pleasantness of eating those foods or the degree of self-control required to NOT eat those foods (Fig. 5A, see “Methods” section for task details). Importantly, while both task conditions involved hedonic judgments about foods (implicitly or explicitly), the Self-Control condition also required participants to simulate inhibitory control of food consumption. As expected, we identified that the average ratings of foods during the Pleasantness and Self-Control conditions were nearly perfectly correlated (*r*(41) = 0.99, *p* < 0.001; Fig. 5B), and the average correlation of those ratings across participants was 0.90 (SD = 0.16).

We analyzed the fMRI data from this task using a regression model which incorporated participants’ ratings, in order to examine the degree to which the response to food pictures was modulated by those pleasantness and self-control inferences. We performed a 2-factor ANOVA to compare the beta coefficients resulting from this model across food networks and across task conditions. We observed a significant effect of network (*F*(1,14) = 25.5, *p* < 0.001), a significant effect of condition (*F*(1,1285) = 28.2, *p* < 0.001), and a significant network-by-condition interaction (*F*(1,1285) = 26.4, *p* < 0.001). Post hoc mixed-effects *t*-tests identified that the rating-modulated response in the Prefrontal network was significantly greater during the Self-Control than during the Pleasantness condition (P – SC: *t*(539) = −6.7, *p* < 0.001; Table S4), while the Limbic network response did not differ between Pleasantness and Self-Control conditions (P – SC: *t*(705) = −0.66, *p* = 0.54; Table S4, Fig. 5A). Thus, the responses of the Prefrontal network were sensitive to the shift in context between conditions and were strongly modulated by the inferred degree of self-control required to inhibit food consumption. Whereas the Limbic network was insensitive to the contextual shift and was primarily modulated by participants’ hedonic judgments of the foods.

**Fig. 6:**
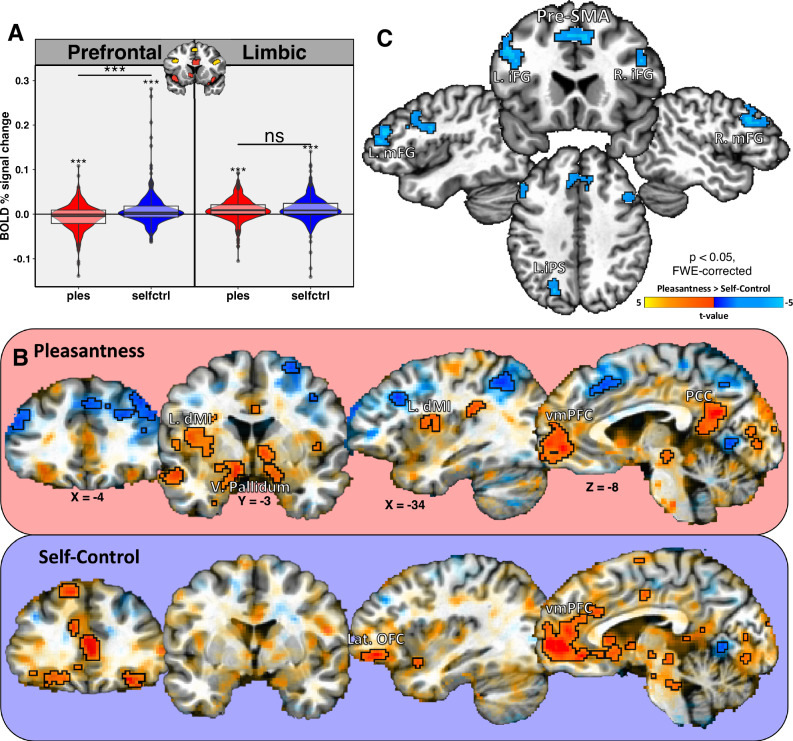
Prefrontal and limbic food networks exhibit differential relationships across PSC task conditions. We examined how the neural response to food images in the PSC task was modulated by participants’ ratings. **A** Neural responses within the Prefrontal network were positively related to ratings during the Self-Control condition, and negatively related to ratings during the Pleasantness condition. In contrast, neural responses within the Limbic network were positively related to ratings during both the Pleasantness and Self-Control conditions. Error bars represent the standard error of the mean. Boxplot lines signify distribution medians. Boxplot bottoms are distribution lower quartiles, and tops are upper quartiles. **B** Whole-brain analyses of both task conditions identified a number of limbic cortical and sub-cortical brain regions whose response to food pictures was positively related to ratings, though some regions such as the mid-insula, ventral pallidum, and lateral OFC were more active during one condition or another. NB: In this panel, FWE-corrected brain regions are ‘highlighted’, and regions that did not meet the statistical thresholds are shown for illustrative purposes only, to demonstrate the similarity of response profiles across conditions^53^. **C** A whole-brain analysis contrasting the task conditions specifically identified regions of the prefrontal and parietal cortex whose rating-modulated response to food pictures was significantly greater during the Self-Control condition. vmPFC – ventromedial Prefrontal Cortex. PCC – Posterior Cingulate Cortex.

We confirmed these findings with whole-brain analyses which identified a number of brain regions whose response to food pictures was positively related to participants’ ratings during the pleasantness and self-control conditions (Fig. 6B). Regions positively associated with both Pleasantness and Self-Control included reward-associated regions such as the bilateral orbitofrontal cortex and ventral striatum, along with other regions such as the ventromedial prefrontal cortex (Tables S5 and S6). Some regions, such as the bilateral dorsal mid-insula, ventral pallidum, and posterior cingulate cortex, were identified within the pleasantness but not the self-control contrast. Other regions such as more lateral areas of OFC, were observed in the self-control and not the pleasantness contrast (Fig. 6B, Table S6). Finally, we contrasted the two conditions at the whole-brain level, and we observed multiple regions within the prefrontal and parietal cortex, including the IFG, MFG, IPS, and Pre-SMA, whose response was significantly greater during the Self-Control than during the Pleasantness condition (Fig. 6C, Table S7).

### Supplemental analyses for effects of BMI, hunger, and satiety

We performed a series of supplemental analyses to examine whether the Body-Mass-Index (BMI) of participants or their ratings of hunger or satiety before scan sessions had a significant effect on our neuroimaging results. At the univariate level, we observed no significant relationship between the participants’ BMI, Hunger, or Satiety ratings and the response to food vs. objects pictures, after statistical thresholding and correction for multiple comparisons. At the multivariate level, we did not identify a significant effect of BMI, Hunger, or Satiety (all *p*’s ≤ 0.25) or any significant factor-by-network interactions in our RSA ROI analyses (*p* ≤ 0.51). In our whole-brain analyses of the PSC task data, we also did not observe any brain regions exhibiting a significant relationship with participant BMI, Hunger, or Satiety.

## Discussion

The present study was designed to examine how fine-grained food-property information is represented in the brain. To accomplish this, we examined the hemodynamic response to pictures of 36 foods (and non-food objects), shown to subjects during fMRI as they performed an orthogonal repetition-detection task. We identified a core set of brain regions frequently associated with food responses and then compared the multivariate responses to food images within these regions using a clustering method based on Representational Similarity Analysis (RSA)^15^. This allowed us to identify whether these areas were representing the same type of information about these foods equally, or whether there was some reliable difference in the information represented across these regions. Our clustering analysis identified two sub-networks exhibiting categorically different multivariate responses to food pictures: a Prefrontal network of regions, including areas of the dorsolateral prefrontal cortex (dlPFC), such as the bilateral inferior frontal and medial frontal gyri, the Pre-Supplementary Motor Area (Pre-SMA), as well as an area of the right intraparietal sulcus (IPS); and a Limbic network of regions, including the bilateral insular cortex (mid-insula and ventral anterior insula), orbitofrontal cortex (OFC), amygdala, and ventral striatum. The Prefrontal sub-network regions largely overlap with the canonical Fronto-Parietal and Dorsal Attention Networks identified through widescale analysis of resting-state functional connectivity^24^. These regions have variously been associated with goal-directed attention, behavioral response inhibition, and self-control, and are implicated in the pathophysiology of eating disorders^25–28^, which points towards a role for this Prefrontal network in the behavioral regulation of food consumption. In contrast, the Limbic network regions largely overlap with the canonical Cingulo-Opercular and Somatomotor resting-state networks^24^. As many of the regions composing the Limbic sub-network were previously associated with appetitive processing and the hedonic response to sensory stimuli^29–31^, we reasoned that this sub-network would be relatively more involved in the representation of hedonic information about food.

To test these possibilities, we analyzed the data from a second neuroimaging task, which was designed to dissociate purely hedonic estimates of foods from estimates of the mental effort required to control food consumption. During this task, our participants viewed another set of food images during two task conditions, where they provided ratings of either the expected pleasantness of eating those foods, or the degree of self-control required to NOT eat those foods. Using parameter-modulated regression, we examined the degree to which the neural response to food images during those two task conditions was related to participants’ moment-to-moment ratings. As expected, we observed that the hemodynamic response to food pictures within the Limbic sub-network was positively related to participant ratings during the pleasantness condition. Importantly, it was equally responsive to ratings during the self-control condition, indicating that the response to food pictures in these regions was primarily related to the hedonic value of foods, and was not sensitive to the difference in context between task conditions. In contrast, the response to food pictures in the Prefrontal network of regions was highly sensitive to task context, as it was positively related to participant ratings in the self-control condition but negatively related to those ratings during the pleasantness condition. A contrast of the two conditions at the whole-brain level specifically identified a network of prefrontal and parietal regions exhibiting a significantly greater modulation during the self-control than the pleasantness condition, such as the inferior frontal gyrus, medial frontal gyrus, left intraparietal sulcus, and pre-SMA. The results of this second imaging task suggest that these food-responsive sub-networks are not merely functionally distinct, but antagonist, acting as opponent processes involved in regulating approach and avoidance behavior towards food.

Within these sub-networks, we performed RSA using behavioral data from a recent online study using those food pictures^17^, to examine the information content automatically retrieved from within these distinct food networks. This RSA indicated that neural similarity, primarily in the prefrontal food network, was significantly related to similarity along the first principal component dimension of the food behavioral data. This principal component was itself almost perfectly correlated with subjective ratings of both how processed those foods were as well as how (un)healthful those foods were^17^. This result suggests that, upon viewing images of food, information about the relative degree of food processing is automatically represented within a network of regions we identified as involved in the inhibitory control of food consumption.

These results align with and extend previous research on the valuation and regulation of food-related decision-making. Previous studies of this behavioral domain^26^ have demonstrated that focusing attention on the health attributes of foods can modulate neural activity in the ventromedial prefrontal cortex and dorsolateral prefrontal cortex, leading to healthier food choices. Similarly, our results indicate that the prefrontal network is not only responsive to the healthfulness of foods but is also preferentially engaged when participants simulate inhibitory control to resist eating certain foods. This suggests a broader role for the prefrontal network in integrating health-related and self-control considerations, thereby enabling adaptive decision-making in food-related contexts.

Notably, the lack of association between the Limbic network and the behavioral food similarity matrix suggests that, in the context of the Food Pictures task, this network primarily represents categorical distinctions (e.g., food vs. non-food) rather than fine-grained differences between individual food items. However, the PSC task results show that this network does distinguish between individual food items based on their hedonic value. This suggests that the Limbic network’s responsiveness to food cues is context-dependent, and the distinction between food items may only become apparent in tasks where participants explicitly engage in hedonic judgments or decision-making processes.

We additionally performed a searchlight RSA to identify other regions in the wider brain also representing information related to the healthfulness or artificiality of foods. In addition to the fronto-parietal regions previously identified, we also identified several regions representing this information within the ventral occipitotemporal cortex (VTC). This result is in accordance with studies that identified that the response to food pictures within the VTC could be discriminated on the basis of other food properties, specifically their taste-category^32,33^. This general area of the brain is heavily involved in object perception and recognition (see ref. ^34^ for review) and many of the regions identified in our analysis are frequently associated with the processing of objects and scenes. Accordingly, recent studies have suggested that the VTC exhibits a degree of selectivity for food images^4–6^, reasoning that the ecological importance of food for survival would necessitate a degree of specialization in this area. The present results suggest that this apparent selectivity might be related to the representation of information about the level of food processing or artificiality.

The importance of this processed vs. unprocessed dimension to the conceptual knowledge of food has been suggested by previous behavioral and neuroscientific studies, possibly as an extension of a more general distinction between living/non-living or animate/inanimate objects (see refs. ^17,18,35^ for discussion). Neuropsychological studies have identified patients with naming deficits that are specific to fruits and vegetables and not processed foods^36–38^. Behavioral studies have shown significantly better recognition memory for processed vs. unprocessed foods in healthy participants^39^ and that naming accuracy for processed food is relatively spared (compared to unprocessed foods) in patients with Alzheimer’s Disease^40^, supporting the notion that these food types have distinct representations within memory. EEG studies of visual-evoked responses to food images have identified that the natural/processed food dimension can be identified within the first 200 ms after stimulus onset, suggesting that it is one of the first and most salient food properties recognized by the brain^41,42^. The results of the present study serve to highlight the relevance of this conceptual knowledge of food to eating behavior. In our modern food environment, the inhibitory control of food is primarily relevant in regard to foods that are highly processed, energy-dense, and of limited nutritional value. Thus, the automatic representation of information about these food properties within prefrontal food regions could serve a key function of readying individuals to regulate their behavioral response to foods in the environment, facilitating flexible approach or avoidance responses to foods, based on their perceived nutritional content. Importantly, differential responses to food stimuli in this network, such as those seen in anorexia (heightened) and bulimia nervosa (decreased), in a recent meta-analysis of neuroimaging studies of eating disorders^43^, could reflect differential representation of this food-related information within these clinical populations.

The present findings provide novel insights into the neural mechanisms underlying food-related cognition. Through the application of data-driven analytic techniques, we identified distinct neural networks—limbic and prefrontal—that encode complementary aspects of food representation. Unlike traditional univariate analyses that primarily differentiate food from non-food stimuli, our Representational Similarity Analysis (RSA) and clustering methods revealed a functional dissociation between networks encoding hedonic and regulatory processes. This dual-network organization suggests that food-related decisions are guided by an interplay between the hedonic valuation of stimuli and higher-order regulatory control. Individually, these results demonstrate that the prefrontal network preferentially encodes information related to food healthfulness and processing, likely reflecting its role in supporting self-control and goal-directed behavior. The limbic network, in contrast, is tuned to the hedonic value of food, highlighting its role in automatic, affective responses. Collectively, these findings underscore the importance of integrating multiple analytical approaches to capture the complexity of food-related neural representations. Beyond simply categorizing food vs. non-food stimuli, these results provide a framework for understanding how different types of food-related information are represented, retrieved, and integrated to guide behavior.

## Methods

### Participants

Forty-three healthy, native-English-speaking volunteers from the greater Washington, DC metropolitan area were included in this study (18 female; Age (SD): 31 (7.8). Range: 20–45; BMI (SD): 29.2 (7.9) kg/m^2^. Range: 20–45). All participants were right-handed non-smokers. Participants were excluded from taking part in the study if they had diabetes, or if they reported any recent weight changes (>5 kg) in the past 6 months, any allergies to food or local anesthetics, any involvement in regular vigorous exercise regimens, daily use of alcohol or illicit drugs in the previous 6 months, or any strict dietary concerns (vegetarian or kosher diet). Participants were also excluded if they had any history of neurological injury, known genetic or medical disorders that may impact the results of cognitive testing and/or neuroimaging, prenatal drug exposure, severely premature birth or birth trauma, past or present psychiatric conditions (e.g., depression or anxiety disorders), current usage of psychotropic medications, or any exclusion criteria for MRI. A subset of the neuroimaging data from these participants was included separately in previous studies^20,30,44^. The institutional review board of the National Institutes of Health approved all procedures and written informed consent was obtained for all subjects. All ethical regulations relevant to human research participants were followed.

### Experimental design

All participants were admitted as inpatients to the NIH Clinical Center during scanning days. For at least 72 h prior to inpatient admission, participants were provided a eucaloric standardized, balanced diet (50% carbohydrate, 35% fat, and 15% protein), to stabilize body weight and standardize nutrient input across participants. During their inpatient stay, participants continued this eucaloric diet, with no access to outside food. Each participant’s total caloric intake was based on 3-day diet and activity records, resting metabolic rate, and body size measurements made during subject screening. At noon on the first day of fMRI scanning, each subject was provided one of these meals, which were standardized for macronutrient content. Subjects then entered the scanner at 14:00 during which they received an anatomical MRI scan and performed the Food Pictures fMRI task.

On the second day, subjects ate a standardized meal at 13:30, followed by a blood draw at 17:30 and an fMRI scan at 18:00. During this scan, participants received another structural MRI scan and a series of fMRI scans during which they performed a resting-state task and the Food Pleasantness and Self-Control fMRI task. Immediately before both scan sessions, subjects completed interval scale measures indicating their level of hunger (0 = no hunger, 10 = extremely hungry) and sensations of fullness (0 = not at all full, 10 = very full).

#### Food pictures fMRI task

During this task, subjects viewed a broad selection of photographs of food and non-food objects. Food images included foods high and low in both fat and sugar content (see Fig. 3). The non-food photographs all depicted small, manipulable household and office implements (e.g., hammers, staplers, pliers; *N* = 9). Five exemplar images of each food and non-food object were presented in this task, for a total of 180 food and 45 non-food pictures were presented. The pictures were normed in an earlier study to ensure that food and non-food picture naming accuracy was at the ceiling and typicality ratings were not different between picture categories^20^. All pictures were presented for 2.5 s in a pseudo-random order optimized for fMRI task design using optseq2 (http://surfer.nmr.mgh.harvard.edu/optseq). Images were followed by variable duration interstimulus intervals (ISI; 2.5–12.5 s), during which a black fixation cross appeared against a gray background. Subjects were instructed to press a button when two consecutive pictures contained objects with the same name (for example, two consecutive donut images).

#### Food pleasantness and self-control (PSC) task

During this task, subjects viewed pictures of various types of foods that varied in their palatability, including both high-calorie highly palatable foods and less palatable low-calorie foods (Fig. 5a). In one task condition, subjects were asked to provide ratings of how pleasant it would be to eat the depicted food at the present moment (Pleasantness condition). Subjects made interval scale responses during rating periods using an MR-compatible handheld scroll wheel, on a number line which ranged from 1 “Neutral” to 7 “Extremely Pleasant”. Subjects rated foods that would be unpleasant to eat with an “X”, which was located at the bottom of the number line. In another task condition, subjects rated how much self-control it would take to NOT eat the depicted food at the present moment (Self-Control condition). Subjects rated the amount of self-control required on a number line which ranged from 1 “Little Self-Control” to 7 “Extreme Self-Control”. Food pictures were presented for 5 s each, separated by the presentation of fixation cross during a variable duration interstimulus interval (mean ISI = 3.7 s; duration 2.5–7.5 s). The PSC task used a truncated 7-point scale, instead of the 10-point scale used for hunger and fullness ratings. This was done to provide adequate space for the rating boxes to be displayed clearly on screen and to facilitate participant responses using the handheld scroll wheel, within the 5-s response period. Prior to the start of each 8-min run, a slide was presented for five seconds reminding subjects of the instructions for the following task (i.e., “If given the opportunity right now, how pleasant would it be to eat this food?”).

In total, subjects viewed 3 different exemplars of 48 types of food. These foods ranged from highly palatable, high-calorie foods with high fat and/or sugar content (e.g., cheeseburgers, french fries, pizza, cake, cinnamon rolls, ice cream, etc.) to uncooked fruits and vegetables (grapes, strawberries, cauliflower, broccoli, carrots, etc.). The depicted food items were prepared by laboratory personnel, which allowed for high control of stimulus presentation, lighting, portion size, and a standard background (white plate against a gray backdrop photographed within a photobox). Subjects saw pictures of the same food items during both the pleasantness and self-control portions of the experiment, but the photographs presented were taken from different visual angles, which ensured that the subject never saw the same photograph more than once. The task stimuli used in the present study were independently normed for nameability, typicality, perceived fat, and sugar content (see ref. ^30^ for more details).

Visual stimuli for both tasks were projected onto a screen located inside the scanner bore and viewed through a mirror system mounted on the head coil. Stimulus presentation was controlled using E-Prime 2.0 software (Psychology Software Tools, Pittsburgh, PA).

### fMRI imaging methods

fMRI data was collected at the NIMH fMRI core facility at the NIH Clinical Center, using a General Electric MR750 3-Tesla MRI scanner (GE Healthcare, Milwaukee, Wisconsin) and a Nova 16-channel receive-only head coil. During the Food Pictures task, 139 echoplanar image (EPI) volumes were acquired, which consisted of 44 2.8-mm axial slices (echo time (TE) = 27 ms, repetition time (TR) = 2500 ms, flip angle = 90 degrees, voxel size = 3.4375 × 3.4375 × 2.8 mm). During the PSC task, 206 EPI volumes were acquired per each scan, using identical imaging parameters. High-resolution T1-weighted magnetization-prepared rapid acquisition gradient-echo (MPRAGE) sequences were also collected (TE = 2.7 ms, TR = 7.24 ms, flip angle = 12 degrees, voxel size = 0.937 × 0.937 × 1.2 mm) and used as anatomical reference images. All structural and functional images were collected with a sensitivity encoding (SENSE) factor of 2, used to reduce image collection time (for structural images) or minimize image distortions (in functional images).

#### Image pre-processing

All image pre-processing was performed using AFNI. Pre-processing steps are consistent with previous studies using these task paradigms^20–23^. The first 4 volumes of each EPI time course (the first 3 volumes for the PSC task) were excluded from data analysis to allow the fMRI signal to reach longitudinal equilibrium, and a slice timing correction was then applied to the remaining volumes of each EPI scan. A de-spiking interpolation algorithm (AFNI’s 3dDespike) was also used to remove transient signal spikes from the EPI data. All EPI volumes were then registered to a base EPI volume using a 6-parameter (3 translations, 3 rotations) motion correction algorithm, and the motion estimates were saved for use as regressors in the subsequent statistical analyses. Volume registration and spatial normalization to Talairach space were implemented in the same transformation step, in order to minimize the number of interpolation steps performed on EPI data. The final voxel resolution was 2 × 2 × 2 mm^3^. Following this, smoothing with a 6 mm full width at half maximum Gaussian kernel was performed, and the signal intensity for each EPI volume was normalized to reflect the percent signal change from each voxel’s mean intensity across the time course.

### Online behavioral study

The methods and results of the online behavioral study are described in full detail in ref. ^17^. In brief, we recruited 487 native-English-speaking participants to perform an odd-one-out triplet detection task^18,19^ on the Amazon Mechanical Turk platform. Within this task, participants selected which of three images was the “odd one out” given a set of three food images (triplets). The food images were selected from a set of 36 representative food images of the 36 foods displayed in the Food Pictures task described above (Fig. 3a). The images chosen for the online task were those selected, in a previous study, to be the most typical exemplars of that type of food^20^. Each participant completed 20 unique triplets so that in total, the full set of 7140 possible triplets was sampled at least once. The data from the task were combined to create a 36 × 36 food similarity matrix by calculating, for each pair of foods, the proportion of triplets containing those foods in which neither food was selected as the “odd one out”. Principal Components Analysis (PCA) was applied to this similarity matrix to determine the dimensions accounting for the most variance in this similarity matrix. Subsequently, K-means clustering was applied to the data plotted along the first two principal component dimensions, which accounted for ~80% of the variance in the data, which identified five sub-categories of foods (denoted as ‘fats’, ‘sweets’, ‘starches’, ‘fruits’, and ‘vegetables’; Fig. 3b). Further analysis of the principal component dimensions, paired with separate online estimates of specific food properties, identified that the first PC was most strongly correlated with subjective interval scale ratings of how processed the foods were (*r* = 0.97) and their relative healthfulness (*r* = −0.96). The second PC was most strongly related to subjective estimates of the sugar and fat content of those foods (*r*^2^ = 0.90).

### Statistics and reproducibility

Test of behavioral measures included correlation tests (Pearson’s correlations, performed in R) for associations between participants’ pleasantness and self-control ratings during the PSC task. Group-level tests of fMRI beta parameters utilized one-sample *t*-tests (versus 0) for the Food Pictures task responses and paired *t*-tests when comparing conditions during the PSC task. The sample size for Food Pictures task analyses was *n* = 43, and *n* = 42 for PSC analyses. Statistical tests for ROI analyses were performed in R, using the *lmer* function from the lme4 package, and the FDR procedure was used to correct the results for multiple comparisons^45^. Detailed information on procedures and statistical tests is provided in the specific subsections below.

### Food picture task univariate fMRI analyses

The Food Picture task data task was analyzed at the single-subject level using multiple linear regression models in AFNI’s 3dDeconvolve. The regressors for the food and non-food images in the Food Picture task were modeled using a gamma-variate function beginning at the onset of the picture stimulus from each image. For the standard univariate analysis, two regressors were used, one for all food images and another for images of all non-food objects. Subject-level regression models also included regressors of non-interest to account for each run’s mean, linear, quadratic, and cubic signal trends, as well as the 6 normalized motion parameters (3 translations, 3 rotations) computed during the volume registration pre-processing.

To identify brain regions specifically responsive to food images, a whole-brain random effects, paired-sample *t*-test was performed to compare the hemodynamic response to food images vs. the response to non-food images. An initial p-value threshold of *p* < 0.001 was applied to the statistical map. A cluster-size correction of *p* < 0.05 was implemented using AFNI’s 3dClustsim, separately within a whole-brain mask and via a small-volume correction applied to a sub-cortical mask. The sub-cortical mask was a single contiguous mask created from the union of multiple anatomical regions of interest, which have previously been associated with the response to food pictures^1–3^ including bilateral regions of: the amygdala, hippocampus, corpus striatum, thalamus, and hypothalamus. Masks of these regions were extracted from the DD Desai Maximum Probability Map Atlas, based on probability maps generated for 35 cortical areas^46^ and the parcellation of cortical and sub-cortical structures generated by the FreeSurfer program. This atlas is available in the AFNI software distribution (http://afni.nimh.nih.gov). The combined sub-cortical mask was then dilated by one voxel to fill any gaps between the atlas regions and resampled to the final EPI resolution. With the whole-brain and sub-cortical maps, we used revised versions of AFNI’s 3dFWHMx and 3dClustsim to generate smoothness and cluster-size estimates using a spherical non-Gaussian spatial autocorrelation function, which has been demonstrated to produce corrected cluster-size values approximately equal to those achieved through non-parametric permutation methods^47^.

### Food picture task multivariate fMRI analyses

We used a series of multivariate analyses to examine the representation of food-related information in the brain within regions preferentially responsive to foods (vs. non-food objects). For these analyses, we applied a second subject-level regression model to the data from the Food Pictures task. This model included a separate regressor for each food and non-food object (45 in total), in addition to the regressors of non-interest, described previously. Using the results of the univariate contrast described above, we selected the set of brain regions that exhibited a significant hemodynamic response to food vs. non-food objects (Fig. 1; Table S1). As some of the significant clusters in this analysis spanned multiple anatomical regions that have shown distinct functions in previous studies (e.g., within the insular cortex, see ref. ^48^), we increased the statistical threshold on this contrast until those clusters broke up into multiple sub-clusters. The result of this process created 17 separate food-responsive clusters which we used in the region-of-interest analyses which follow (see Figs. 2 and 3).

#### ROI clustering and network analysis

We performed a network clustering approach based on Representational Similarity Analysis (RSA^15^). Within those regions of interest (ROIs), we used the AFNI program 3dMaskdump to extract the voxel-wise beta coefficients for each of the 36 foods presented during the task, which we imported for further analysis within the R statistical software package (https://www.r-project.org), using custom code and functions (see “Data availability” and “Code availability” sections for links). For each subject and ROI, the output of this process was 36 columns × *N* rows matrix, with *N* being the number of voxels within the ROI. We generated Representational Dissimilarity Matrices (RDMs) for these ROIs by calculating the correlation distance of each pair of columns of these matrices, generating a 36 × 36 dissimilarity matrix. Next, we averaged these RDMs across subjects, in order to generate group average RDMs for each ROI. We then generated a second-level similarity matrix by calculating correlations of the (upper triangular segment of the) RDMs from each pair of ROIs (Fig. 2).

We used a community detection algorithm (the *cluster_optimal* method in the R package *igraph*^49^) to group together brain regions with similar representational profiles. This algorithm finds the community structure which will globally optimize network modularity, relative to all other possible community structures. This clustering procedure was used both as a way of identifying categorical differences in the representation of food-related information in the brain, as well as a method of dimensionality reduction for the analyses which followed. We confirmed the optimal number of clusters for these data using the silhouette method, which involves the calculation of the average silhouette coefficient, a measure of within vs. between cluster similarity, for a range of possible cluster values. The optimal cluster value is thus the value with the largest average silhouette coefficient^50^.

For the following analyses, we excluded the Early Visual Cortex (EVC) ROI, as its representational profile was found to be a clear outlier. Its average multivariate distance from the other ROIs was ~2-2.5 standard deviations greater than the mean distance (scaled Mahalanobis distance *z* = 2.1, scaled correlation distance: *z* = 2.0, scaled Euclidean distance: *z* = 2.5), likely due to its role in representing the low-level visual features of the image stimuli.

#### RSA ROI analyses

Having clustered together our food-responsive ROIs into two distinct networks, we next sought to examine the informational content of these food networks. In particular, we examined whether the information in these food networks reflected the similarity structure we identified in our online behavioral study. To do this, we performed a series of RSAs using the neural RDMs from our food-responsive ROIs and RDMs created from this online behavioral data. The first RSA used an RDM generated from the behavioral food similarity matrix (Fig. 3B). Following this, we performed follow-up RSAs, using RDMs (simple Euclidean distance matrices) generated from the first two Principal Components of the food similarity matrix, to further identify which underlying dimension of this behavioral data best accounted for the neural data in these food networks.

These RSAs were performed by calculating the Spearman rank-order correlations between subject-level neural RDMs and behavioral RDMs. These subject-level correlation coefficients were transformed into *z*-scores through a permutation testing procedure^15^, using a distribution of correlation coefficients generated by randomly shuffling the neural RDMs 10,000 times. We used linear mixed-effects *t*-tests (two-sided) to compare the average z-scores between the separate food ROI networks, and within both networks against zero, using participant and brain region as random factors. Statistical tests were performed in R, using the *lmer* function from the lme4 package, and the FDR procedure was used to correct the results for multiple comparisons^45^.

#### RSA searchlight analyses

We used a searchlight RSA procedure to map the correlation between neural RDMs and target RDMs (the 2 RDMs generated from the first 2 principal components of the online food similarity data) throughout the brain. We performed these searchlight analyses using the CosmoMVPA Matlab toolbox^51^. Using the subject-level regression coefficients for each food item, this process calculated the correlation distance between each pair of foods (as in our ROI analyses above) within each searchlight sphere (radius = 3 voxels) and compared these searchlight neural RDMs to the target RDMs generated from the first 2 principal components of the online behavioral data (in separate analyses). For each searchlight, these comparisons involved calculating the Spearman correlation between neural RDMs and Principal Component RDMs and then recording the Fisher-transformed correlation coefficient at each searchlight center. We combined the subject-level searchlight maps generated by these procedures using a one-sample *t*-test, implemented in AFNI’s 3dttest + +, to identify brain regions showing a significant relationship between neural similarity and target similarity at the group level. We applied an initial FDR-corrected threshold of *p*-FDR < 0.05 to the resulting statistical map and subsequently used AFNI’s 3dClustsim (an updated version employing non-Gaussian spatial autocorrelation estimates) to perform cluster-size correction for multiple comparisons within a whole-brain mask.

### PSC task univariate fMRI analyses

Subject-level (i.e., first-level) regression analyses of the fMRI task data were performed in AFNI’s 3dDeconvolve. The regression model included separate regressors for the Pleasantness and Self-control conditions as well as the instruction slides. These regressors were constructed by convolution of a gamma-variate function with a canonical hemodynamic response function. Importantly, the pleasantness and self-control conditions were modeled using amplitude modulation regression in AFNI, which generates one regressor for image onset and one additional regressor in which the height of the hemodynamic response varies with the height of the associated behavioral covariate. In this case, the predicted response to food picture presentation varied (linearly) as a function of subjects’ pleasantness or self-control ratings. These regressors thus identified brain regions where the response to food pictures was modulated by subjects’ moment-to-moment inferences of the pleasantness or self-control associated with specific foods. Additional regressors were included for the instruction slide times, any missed response periods, and any food pictures that the subject indicated were unpleasant. Finally, the regression model also included regressors of non-interest to account for each run’s mean, linear, quadratic, and cubic signal trends, as well as the 6 normalized motion parameters (3 translations, 3 rotations) computed during the volume registration pre-processing.

We used the AFNI program 3dROIstats to extract the average amplitude-modulated beta coefficients for the PSC task within the food-responsive ROIs defined previously, which we imported for further analysis within the R software package. We performed a task-by-condition ANOVA to compare those beta coefficients within the food networks defined in the previous analyses, followed by *post hoc* linear mixed-effects *t*-tests, as above, to examine the ANOVA results. These analyses thus examined how much the response to food pictures in those networks was modulated by subjects’ pleasantness or self-control ratings. We also performed a whole-brain analysis using AFNI’s 3dttest + + contrasting the pleasantness and self-control conditions, which identified brain regions where the hemodynamic response to food pictures showed a greater modulation by either pleasantness or self-control ratings. We applied an initial threshold of *p* < 0.005 to the resulting statistical map and AFNI’s 3dClustsim (with non-Gaussian spatial autocorrelation estimates) to perform cluster-size correction for multiple comparisons within a whole-brain mask.

### Supplemental analyses for effects of BMI

We additionally examined whether the neural response to food pictures during our tasks was related to the BMI of our participants. We also included BMI as a covariate in the RSAs using the principal components of the food similarity matrix. At the whole-brain level, each whole-brain analysis performed in this study included BMI as a participant-level covariate using AFNI’s 3dttest++. This process allowed us to both examine the effect of BMI on our condition of interest and regress out that effect in a single step.

### Reporting summary

Further information on research design is available in the Nature Portfolio Reporting Summary linked to this article.

## Supplementary information

Supplementary Information

Reporting Summary

## Data Availability

The statistical summary data and anonymized anatomical and fMRI data for the current study^52^ have been placed in a public repository: 10.17605/OSF.IO/8DS7G. All other relevant files are available upon reasonable request.

## Code availability

The analysis code for the current study has been placed in a public repository^52^: 10.17605/OSF.IO/8DS7G.

## References

[1] van Meer, F., van der Laan, L. N., Adan, R. A., Viergever, M. A. & Smeets, P. A. What you see is what you eat: an ALE meta-analysis of the neural correlates of food viewing in children and adolescents. *NeuroImage* **104**, 35–43 (2015). 25285373 10.1016/j.neuroimage.2014.09.069

[2] Tang, D. W., Fellows, L. K., Small, D. M. & Dagher, A. Food and drug cues activate similar brain regions: a meta-analysis of functional MRI studies. *Physiol. Behav.* **106**, 317–324 (2012). 22450260 10.1016/j.physbeh.2012.03.009

[3] Laan, L. N., van der, Ridder, D. T. D., de, Viergever, M. A. & Smeets, P. A. M. The first taste is always with the eyes: a meta-analysis on the neural correlates of processing visual food cues. *NeuroImage* **55**, 296–303 (2011). 21111829 10.1016/j.neuroimage.2010.11.055

[4] Khosla, M., Murty, N. A. R. & Kanwisher, N. A highly selective response to food in human visual cortex revealed by hypothesis-free voxel decomposition. *Curr. Biol.* **32**, 4159–4171.e9 (2022). 36027910 10.1016/j.cub.2022.08.009 PMC9561032

[5] Pennock, I. M. L. et al. Color-biased regions in the ventral visual pathway are food selective. *Curr. Biol.* **33**, 134–146.e4 (2023). 36574774 10.1016/j.cub.2022.11.063 PMC9976629

[6] Jain, N. et al. Selectivity for food in human ventral visual cortex. *Commun. Biol.* **6**, 175 (2023). 36792693 10.1038/s42003-023-04546-2 PMC9932019

[7] LaBar, K. S. et al. Hunger selectively modulates corticolimbic activation to food stimuli in humans. *Behav. Neurosci.* **115**, 493–500 (2001). 11345973 10.1037/0735-7044.115.2.493

[8] Simmons, W. K., Martin, A. & Barsalou, L. W. Pictures of appetizing foods activate gustatory cortices for taste and reward. *Cereb. Cortex* **15**, 1602–1608 (2005). 15703257 10.1093/cercor/bhi038

[9] Downing, P. E., Chan, A. W.-Y., Peelen, M. V., Dodds, C. M. & Kanwisher, N. Domain specificity in visual cortex. *Cereb. Cortex* **16**, 1453–1461 (2006). 16339084 10.1093/cercor/bhj086

[10] Siep, N. et al. Hunger is the best spice: an fMRI study of the effects of attention, hunger and calorie content on food reward processing in the amygdala and orbitofrontal cortex. *Behav. Brain Res.* **198**, 149–158 (2009). 19028527 10.1016/j.bbr.2008.10.035

[11] DiFeliceantonio, A. G. et al. Supra-additive effects of combining fat and carbohydrate on food reward. *Cell Metab.* **28**, 33–44.e3 (2018). 29909968 10.1016/j.cmet.2018.05.018

[12] Neumark-Sztainer, D., Story, M., Perry, C. & Casey, M. A. Factors influencing food choices of adolescents findings from focus-group discussions with adolescents. *J. Am. Diet. Assoc.* **99**, 929–937 (1999). 10450307 10.1016/S0002-8223(99)00222-9

[13] Finlayson, G., King, N. & Blundell, J. E. Is it possible to dissociate ‘liking’ and ‘wanting’ for foods in humans? A novel experimental procedure. *Physiol. Behav.* **90**, 36–42 (2007). 17052736 10.1016/j.physbeh.2006.08.020

[14] Hall, K. D. et al. Ultra-processed diets cause excess calorie intake and weight gain: an inpatient randomized controlled trial of ad libitum food intake. *Cell Metab.* **32**, 690 (2020). 33027677 10.1016/j.cmet.2020.08.014

[15] Kriegeskorte, N., Mur, M. & Bandettini, P. Representational similarity analysis – connecting the branches of systems neuroscience. *Front. Syst. Neurosci.* **2**, 4 (2008). 19104670 10.3389/neuro.06.004.2008 PMC2605405

[16] Mur, M., Bandettini, P. A. & Kriegeskorte, N. Revealing representational content with pattern-information fMRI—an introductory guide. *Soc. Cogn. Affect. Neurosci.* **4**, 101–109 (2009). 19151374 10.1093/scan/nsn044 PMC2656880

[17] Carrington, M., Liu, A. G., Candy, C., Martin, A. & Avery, J. A. Naturalistic food categories are driven by subjective estimates rather than objective measures of food qualities. *Food Qual. Prefer.* **113**, 105073 (2024). 38222065 10.1016/j.foodqual.2023.105073 PMC10783799

[18] Hebart, M. N., Zheng, C. Y., Pereira, F. & Baker, C. I. Revealing the multidimensional mental representations of natural objects underlying human similarity judgements. *Nat. Hum. Behav.* **4**, 1173–1185 (2020). 33046861 10.1038/s41562-020-00951-3 PMC7666026

[19] Avery, J. A., Liu, A. G., Carrington, M. & Martin, A. Taste metaphors ground emotion concepts through the shared attribute of valence. *Front. Psychol.* **13**, 938663 (2022). 35903735 10.3389/fpsyg.2022.938663 PMC9314637

[20] Simmons, W. K. et al. Category-specific integration of homeostatic signals in caudal but not rostral human insula. *Nat. Neurosci.* **16**, 1551–1552 (2013). 24077565 10.1038/nn.3535 PMC3835665

[21] Simmons, W. K. et al. Appetite changes reveal depression subgroups with distinct endocrine, metabolic, and immune states. *Mol. Psychiatry* **25**, 1457–1468 (2020). 29899546 10.1038/s41380-018-0093-6 PMC6292746

[22] Avery, J. A. et al. Neural correlates of taste reactivity in autism spectrum disorder. *NeuroImage Clin.* **19**, 38–46 (2018). 30035000 10.1016/j.nicl.2018.04.008 PMC6051474

[23] Simmons, W. K. et al. Depression-related increases and decreases in appetite: dissociable patterns of aberrant activity in reward and interoceptive neurocircuitry. *Am. J. Psychiatry* **173**, 418–428 (2016). 26806872 10.1176/appi.ajp.2015.15020162 PMC4818200

[24] Yeo, B. T. T. et al. The organization of the human cerebral cortex estimated by intrinsic functional connectivity. *J. Neurophysiol.* **106**, 1125–1165 (2011). 21653723 10.1152/jn.00338.2011 PMC3174820

[25] Hampshire, A., Chamberlain, S. R., Monti, M. M., Duncan, J. & Owen, A. M. The role of the right inferior frontal gyrus: inhibition and attentional control. *NeuroImage* **50**, 1313–1319 (2010). 20056157 10.1016/j.neuroimage.2009.12.109 PMC2845804

[26] Hare, T. A., Malmaud, J. & Rangel, A. Focusing attention on the health aspects of foods changes value signals in vmPFC and improves dietary choice. *J. Neurosci.* **31**, 11077–11087 (2011). 21795556 10.1523/JNEUROSCI.6383-10.2011 PMC6623079

[27] Roberts, G. et al. Reduced inferior frontal gyrus activation during response inhibition to emotional stimuli in youth at high risk of bipolar disorder. *Biol. Psychiatry* **74**, 55–61 (2013). 23245750 10.1016/j.biopsych.2012.11.004

[28] Kaye, W. H., Wierenga, C. E., Bailer, U. F., Simmons, A. N. & Bischoff-Grethe, A. Nothing tastes as good as skinny feels: the neurobiology of anorexia nervosa. *Trends Neurosci.* **36**, 110–120 (2013). 23333342 10.1016/j.tins.2013.01.003 PMC3880159

[29] Klein-Flügge, M. C., Barron, H. C., Brodersen, K. H., Dolan, R. J. & Behrens, T. E. J. Segregated encoding of reward–identity and stimulus–reward associations in human orbitofrontal cortex. *J. Neurosci.* **33**, 3202–3211 (2013). 23407973 10.1523/JNEUROSCI.2532-12.2013 PMC3586675

[30] Simmons, W. K. et al. The ventral pallidum and orbitofrontal cortex support food pleasantness inferences. *Brain Struct. Funct.* **219**, 473–483 (2014). 23397317 10.1007/s00429-013-0511-0 PMC3676475

[31] Berridge, K. C. & Kringelbach, M. L. Pleasure systems in the brain. *Neuron* **86**, 646–664 (2015). 25950633 10.1016/j.neuron.2015.02.018 PMC4425246

[32] Avery, J. A., Carrington, M. & Martin, A. A common neural code for representing imagined and inferred tastes. *Prog. Neurobiol.* **223**, 102423 (2023). 36805499 10.1016/j.pneurobio.2023.102423 PMC10040442

[33] Avery, J. A., Liu, A. G., Ingeholm, J. E., Gotts, S. J. & Martin, A. Viewing images of foods evokes taste quality-specific activity in gustatory insular cortex. *Proc. Natl Acad. Sci. USA* **118**, e2010932118 (2021). 33384331 10.1073/pnas.2010932118 PMC7812770

[34] Kravitz, D. J., Saleem, K. S., Baker, C. I., Ungerleider, L. G. & Mishkin, M. The ventral visual pathway: an expanded neural framework for the processing of object quality. *Trends Cogn. Sci.* **17**, 26–49 (2013). 23265839 10.1016/j.tics.2012.10.011 PMC3532569

[35] Capitani, E., Laiacona, M., Mahon, B. & Caramazza, A. What are the facts of semantic category-specific deficits? A critical review of the clinical evidence. *Cogn. Neuropsychol.* **20**, 213–261 (2003). 20957571 10.1080/02643290244000266

[36] Hart, J., Berndt, R. S. & Caramazza, A. Category-specific naming deficit following cerebral infarction. *Nature* **316**, 439–440 (1985). 4022134 10.1038/316439a0

[37] Crutch, S. J. & Warrington, E. K. The selective impairment of fruit and vegetable knowledge: amultiple processing channels account of fine-grain category specificity. *Cogn. Neuropsychol.* **20**, 355–372 (2003). 20957575 10.1080/02643290244000220

[38] Renzi, E. D. & Lucchelli, F. Are semantic systems separately represented in the brain? The case of living category impairment. *Cortex* **30**, 3–25 (1994). 8004989 10.1016/s0010-9452(13)80322-x

[39] Aiello, M. et al. Episodic memory for natural and transformed food. *Cortex* **107**, 13–20 (2018). 29843896 10.1016/j.cortex.2018.04.013

[40] Rumiati, R. I., Foroni, F., Pergola, G., Rossi, P. & Silveri, M. C. Lexical-semantic deficits in processing food and non-food items. *Brain Cogn.* **110**, 120–130 (2016). 27651170 10.1016/j.bandc.2016.08.007

[41] Coricelli, C., Toepel, U., Notter, M., Murray, M. M. & Rumiati, R. I. Distinct brain representations of processed and unprocessed foods. *Eur. J. Neurosci.* **50**, 3389–3401 (2019). 31228866 10.1111/ejn.14498

[42] Moerel, D., Psihoyos, J. & Carlson, T. A. The time-course of food representation in the human brain. *J. Neurosci.* **44**, e1101232024 (2024). 38740441 10.1523/JNEUROSCI.1101-23.2024 PMC11211715

[43] Bronleigh, M., Baumann, O. & Stapleton, P. Neural correlates associated with processing food stimuli in anorexia nervosa and bulimia nervosa: an activation likelihood estimation meta-analysis of fMRI studies. *Eat. Weight Disord. Stud. Anorex., Bulim. Obes.* **27**, 2309–2320 (2022). 10.1007/s40519-022-01390-x PMC9556419 35304713

[44] Darcey, V. L. et al. Dietary fat restriction affects brain reward regions in a randomized crossover trial. *JCI Insight* **8**, e169759 (2023). 37345661 10.1172/jci.insight.169759 PMC10371234

[45] Benjamini, Y. & Hochberg, Y. Controlling the false discovery rate: a practical and powerful approach to multiple testing. *J. R. Stat. Soc. Ser. B Methodol.* **57**, 289–300 (1995).

[46] Desikan, R. S. et al. An automated labeling system for subdividing the human cerebral cortex on MRI scans into gyral based regions of interest. *NeuroImage* **31**, 968–980 (2006). 16530430 10.1016/j.neuroimage.2006.01.021

[47] Cox, R. W., Chen, G., Glen, D. R., Reynolds, R. C. & Taylor, P. A. fMRI clustering and false-positive rates. *Proc. Natl Acad. Sci. USA* **114**, E3370–E3371 (2017). 28420798 10.1073/pnas.1614961114 PMC5410825

[48] Kurth, F., Zilles, K., Fox, P. T., Laird, A. R. & Eickhoff, S. B. A link between the systems: functional differentiation and integration within the human insula revealed by meta-analysis. *Brain Struct. Funct.* **214**, 519–534 (2010). 20512376 10.1007/s00429-010-0255-z PMC4801482

[49] Kolaczyk, E. D. & Csárdi, G. *Statistical Analysis of Network Data with R* 87–113 10.1007/978-3-030-44129-6_6 (2020).

[50] Rousseeuw, P. J. Silhouettes: a graphical aid to the interpretation and validation of cluster analysis. *J. Comput. Appl. Math.* **20**, 53–65 (1987).

[51] Oosterhof, N. N., Connolly, A. C. & Haxby, J. V. CoSMoMVPA: multi-modal multivariate pattern analysis of neuroimaging data in Matlab/GNU octave. *Front. Neuroinform.* **10**, 27 (2016). 27499741 10.3389/fninf.2016.00027 PMC4956688

[52] Avery, J. – FoodSimilarity [Data set]. *Open Science Foundation*10.17605/OSF.IO/8DS7G (2023).

[53] Taylor, P. A. et al. Highlight results, don’t hide them: enhance interpretation, reduce biases and improve reproducibility. *NeuroImage* **274**, 120138 (2023). 37116766 10.1016/j.neuroimage.2023.120138 PMC10233921

