# Supplementary material for: Automatic engagement of limbic and prefrontal networks in response to food images reflects distinct information about food hedonics and inhibitory control
Source: Commun Biol. 2025 Feb 20;8:270. doi: 10.1038/s42003-025-07704-w (PMC11842766; doi:10.1038/s42003-025-07704-w)
Supplement: Supplementary file 1 — Supplementary Information [file 42003_2025_7704_MOESM1_ESM.pdf]

Supplementary Information for

Automatic engagement of limbic and prefrontal networks in response to food images reflects distinct information about food hedonics and inhibitory control

Jason A. Avery[1]*, Madeline Carrington[1], John E. Ingeholm[1], Valerie Darcey[2], W. Kyle Simmons[3], Kevin D. Hall[2], and Alex Martin[1]

1. Laboratory of Brain and Cognition, National Institute of Mental Health, Bethesda, MD, United States 20892

2. Integrative Physiology Section, National Institute of Diabetes & Digestive & Kidney Diseases, Bethesda, MD, United States 20892

3. Department of Pharmacology and Physiology, Oklahoma State University Center for Health Sciences, Tulsa, OK, 74107

*Corresponding Author

Email: jason.avery@nih.gov

This PDF file includes:

Supplemental Results
Tables S1 to S5

Supplemental Results

Food vs. Non-food Searchlight RSA: We performed a searchlight RSA to identify regions exhibiting high neural similarity between all foods vs. all non-food objects. This analysis involved constructing two model RDMs: A food RDM where all edges between foods were 0 and all edges between objects were set to 1 (as well as edges between foods and objects), and an objects RDM where all edges between objects were 0 and all other edges were set to 1. We performed separate searchlight RSAs using the food and object RDMs, as outlined in the Methods (RSA Searchlight Analyses). We combined the subject-level searchlight maps generated by these procedures using a two-sample paired t-test, implemented in AFNI's 3dttest++, to identify brain regions exhibiting a significant difference in representational similarity to the food vs object RDMs. After applying a moderate statistical threshold ($p < 0.005$ voxelwise), we observed some brain regions we had previously identified in the univariate analysis, such as the bilateral dorsal mid-insula and the left lateral occipital cortex, that exhibited a significant relationship between neural similarity and similarity to the food or object RDMs, respectively. However, none of these regions survived cluster-size correction for multiple comparisons at the group level.

Table S1: Brain regions responsive to food and non-food images

Anatomical Location	Peak location			Peak T-value	Peak Beta Coefficient	Volume, mm3	Cluster P-Value
**Food > Non-food Pictures**	X	Y	Z				
Early Visual Cortex	-11	-89	2	13.1	1.62	43344	<< 0.01
L Dorsal Anterior Cingulate Cortex	-3	-61	8	4.73	0.38	3744	<< 0.01
R Mid-Insula	37	1	-4	6.49	0.35	2632	<< 0.01
L Orbitofrontal Cortex (BA11m)	-21	35	-12	6.94	0.80	2416	<< 0.01
R Inferior Frontal Gyrus	41	3	26	6.73	0.34	1784	<< 0.01
R Medial Frontal Gyrus	35	33	10	6.48	0.32	1624	<< 0.01
L Ventral Anterior Insula	-39	5	-4	5.15	0.31	1296	<< 0.01
L Inferior Frontal Gyrus	-39	5	28	5.7	0.29	1200	< 0.01
L Pre-Supplementary Motor Area	3	15	42	4.34	0.36	1144	< 0.01
L Medial Frontal Gyrus	-35	33	16	5.33	0.32	1016	< 0.01
L Dorsal Mid-Insula	-33	-5	8	6.28	0.28	944	< 0.01
L Amygdala	-15	3	-16	4.99	0.55	928	< 0.01
R Postcentral Gyrus	59	-15	24	5.38	0.28	824	< 0.01
R Orbitofrontal Cortex (BA11m)	21	31	-16	5.25	0.70	784	< 0.01
R Intraparietal Sulcus	43	-31	34	4.47	0.19	704	< 0.01
L Ventral Striatum	-7	7	2	4.04	0.27	480	< 0.04
R Amygdala	19	-1	-10	4.26	0.42	272	< 0.05
**Non-food > Food Pictures**							
L Lateral Occipital Cortex	-43	-61	4	-7.98	-0.36	9608	<< 0.01
R Sup. Temporal Gyrus<br>R Lateral Occipital Cortex	41	-61	4	-6.85	-0.21	4448	<< 0.01
L Cuneus	5	-71	22	-4.12	-0.27	608	< 0.02

Table S2: Behavioral RSA Network ROI Analyses

Network	RSA Type	DoF	RSA Mean Z-score	SE	t-value	p-value	Diff (Prefrontal vs. Limbic)	T	DoF	P
Prefrontal	FSM*	42	0.213	0.078	2.740	0.003	0.210	2.166	14	0.031
Limbic	FSM*	42	0.003	0.067	0.045	0.482				
Prefrontal	PC1	42	0.500	0.105	4.760	<0.001	0.375	3.030	14	0.003
Limbic	PC1	42	0.124	0.072	1.714	0.088				
Prefrontal	PC2	42	-0.096	0.082	-1.175	0.24	0.007	0.072	14	0.943
Limbic	PC2	42	-0.104	0.063	-1.663	0.10				

*Food Similarity Matrix

Table S3: Brain regions exhibiting a significant relationship between neural similarity to food images and behavioral similarity of foods (PC1 RDM)

Anatomical Location	Peak location			Peak T-value	Peak Correlation Coefficient (Z-scored)	Volume, mm3	Cluster P-Value
	X	Y	Z				
L Middle Occipital Gyrus							
L Lateral Occipital Cortex							
L Parahippocampal Gyrus							
L Fusiform Gyrus	-25	-77	-12	7.82	0.07	37016	<< 0.01
R Middle Occipital Gyrus							
R Lateral Occipital Cortex							
R Parahippocampal Gyrus							
R Fusiform Gyrus	17	-89	-4	6.47	0.06	35560	<< 0.01
L Inferior Frontal Gyrus							
L Middle Frontal Gyrus	-37	-1	26	4.84	0.05	7224	<< 0.01
R Inferior Frontal Gyrus	47	21	18	4.20	0.04	1944	< 0.05
R Middle Frontal Gyrus	45	7	44	4.57	0.04	1896	< 0.05

Table S4: Pleasantness (P) vs. Self-Control (SC) Network ROI Analyses

Network	Task Condition	DoF	Beta Coeff.	SE	t-value	p-value	Diff (P vs. SC)	T	DoF	P
Prefrontal	P	41	-0.006	0.003	-1.99	0.053	-0.016	-6.729	539	<0.001
Prefrontal	SC	41	0.010	0.005	1.99	0.054				
Limbic	P	41	0.010	0.003	3.63	<0.001	-0.001	-0.664	705	0.507
Limbic	SC	41	0.011	0.003	4.00	<0.001				

Table S5. Brain regions exhibiting a significant modulation of the hemodynamic response to food pictures by pleasantness ratings

Anatomical Location	Peak location			Peak T-value	Peak Beta Coefficient	Volume, mm^3	Cluster P-Value
**Pleasantness Modulation - Positive**	x	y	z				
R Early Visual Cortex	7	-83	18	10.79	0.10	10776	<< 0.01
Ventromedial Prefrontal Cortex	-1	51	6	5.19	0.05	5088	<< 0.01
L Putamen							
L Amygdala							
L Ventral Pallidum	-25	-13	-6	5.27	0.04	3968	<< 0.01
L Posterior Cingulate Cortex	-11	-51	28	4.96	0.03	2608	<< 0.01
L Dorsal Mid-Insular Cortex							
L Caudate	-41	7	12	4.92	0.02	2256	<< 0.01
L Secondary Somatosensory Cortex	-41	-27	20	4.99	0.02	1992	<< 0.01
L Superior Temporal Sulcus	-49	-11	-10	4.51	0.02	1240	< 0.01
R Cerebellum	23	-41	-24	4.87	0.02	992	< 0.01
R Dorsal Mid-Insular Cortex	39	3	10	4.15	0.02	800	< 0.03
R Putamen	7	-3	4	4.45	0.03	728	< 0.04
**Pleasantness Modulation - Negative**							

R Middle Frontal Gyrus	39	25	38	-5.19	-0.04	8896	<< 0.01
R Middle Occipital Gyrus	9	-69	0	-10.74	-0.09	6440	<< 0.01
R Precuneus	5	-59	44	-6.53	-0.07	6024	<< 0.01
L Inferior Frontal Gyrus	-55	21	22	-4.87	-0.03	3240	<< 0.01
L Pre-Supplementary Motor Area	5	19	42	-4.42	-0.03	2880	<< 0.01
R Superior Frontal Gyrus	25	-1	58	-4.63	-0.05	912	< 0.02

Table S6. Brain regions exhibiting a significant modulation of the hemodynamic response to food pictures by self-control ratings

**Anatomical Location**	**Peak location**			**Peak T-value**	**Peak Beta Coefficient**	**Volume, mm^3**	**Cluster P-Value**
**Self-Control Modulation - Positive**	x	y	z				
Ventromedial Prefrontal Cortex	3	33	12	8.07	0.05	12400	<< 0.01
R Early Visual Cortex	15	-89	16	9.09	0.10	7048	<< 0.01
L Orbitofrontal Cortex	-37	39	-6	4.91	0.04	1856	< 0.01
R Thalamus	1	-17	0	4.24	0.05	1368	< 0.01
L Superior Frontal Gyrus	-13	29	48	5.32	0.04	1240	< 0.01
L Early Visual Cortex	-9	-79	0	5.62	0.05	1224	< 0.01
L Brain Stem	1	-29	-22	5.07	0.03	1168	< 0.01
R Orbitofrontal Cortex	29	31	-10	5.12	0.04	936	< 0.02
R Hippocampus	23	-27	-10	4.05	0.03	912	< 0.02
L Ventral Anterior Insula	-27	13	-20	4.36	0.05	856	< 0.02
R Fusiform Gyrus	39	-41	-18	4.06	0.03	768	< 0.04
**Self-Control Modulation - Negative**							
R Middle Occipital Gyrus	9	-67	0	-9.41	-0.09	5376	<< 0.01

Table S7. Brain regions exhibiting a difference in modulation by *Pleasantness vs. Self-Ctrl ratings*

**Anatomical Location**	**Peak location**			**Peak T-value**	**Peak Beta Coefficient**	**Volume, mm^3**	**Cluster P-Value**
**Pleasantness vs. Self-Control Modulation**	x	y	z				
L Pre-Supplementary Motor Area	-9	21	38	-4.26	-0.02	2800	<< 0.01
L Inferior Frontal Gyrus	-45	17	30	-4.34	-0.04	2360	<< 0.01
R Inferior Frontal Gyrus	45	19	26	-3.95	-0.03	2216	<< 0.01
R Middle Frontal Gyrus	41	37	22	-4.50	-0.04	1792	< 0.01
L Intraparietal Sulcus	-21	-69	36	-3.73	-0.02	888	< 0.02
