# Supplementary material for: Automatic engagement of limbic and prefrontal networks in response to food images reflects distinct information about food hedonics and inhibitory control
Source: Commun Biol. 2025 Feb 20;8:270. doi: 10.1038/s42003-025-07704-w (PMC11842766; doi:10.1038/s42003-025-07704-w)
Supplement: Supplementary file 2 — Reporting Summary [file 42003_2025_7704_MOESM2_ESM.pdf]

nature portfolio

Corresponding author(s): Jason Avery, PhD

Last updated by author(s): 12/5/2024

Reporting Summary

Nature Portfolio wishes to improve the reproducibility of the work that we publish. This form provides structure for consistency and transparency in reporting. For further information on Nature Portfolio policies, see our Editorial Policies and the Editorial Policy Checklist.

Statistics

For all statistical analyses, confirm that the following items are present in the figure legend, table legend, main text, or Methods section.

n/a	Confirmed	
☐	☒	The exact sample size (*n*) for each experimental group/condition, given as a discrete number and unit of measurement
☐	☒	A statement on whether measurements were taken from distinct samples or whether the same sample was measured repeatedly
☐	☒	The statistical test(s) used AND whether they are one- or two-sided<br>*Only common tests should be described solely by name; describe more complex techniques in the Methods section.*
☐	☒	A description of all covariates tested
☐	☒	A description of any assumptions or corrections, such as tests of normality and adjustment for multiple comparisons
☐	☒	A full description of the statistical parameters including central tendency (e.g. means) or other basic estimates (e.g. regression coefficient) AND variation (e.g. standard deviation) or associated estimates of uncertainty (e.g. confidence intervals)
☐	☒	For null hypothesis testing, the test statistic (e.g. *F*, *t*, *r*) with confidence intervals, effect sizes, degrees of freedom and *P* value noted<br>*Give P values as exact values whenever suitable.*
☒	☐	For Bayesian analysis, information on the choice of priors and Markov chain Monte Carlo settings
☒	☐	For hierarchical and complex designs, identification of the appropriate level for tests and full reporting of outcomes
☐	☒	Estimates of effect sizes (e.g. Cohen's *d*, Pearson's *r*), indicating how they were calculated

Our web collection on statistics for biologists contains articles on many of the points above.

Software and code

Policy information about availability of computer code

Data collection	E-prime software was used for visual stimulus presentation. This is listed in the methods section.
Data analysis	AFNI was used for neuroimaging pre-processing and analyses, and R-studio was used for subsequent statistical analyses. The specific programs used for analysis are listed in the methods section.

For manuscripts utilizing custom algorithms or software that are central to the research but not yet described in published literature, software must be made available to editors and reviewers. We strongly encourage code deposition in a community repository (e.g. GitHub). See the Nature Portfolio guidelines for submitting code & software for further information.

Data

Policy information about availability of data

All manuscripts must include a data availability statement. This statement should provide the following information, where applicable:

- Accession codes, unique identifiers, or web links for publicly available datasets
- A description of any restrictions on data availability
- For clinical datasets or third party data, please ensure that the statement adheres to our policy

The statistical summary data, analysis code, and anonymized anatomical and fMRI data for the current study have been placed in a public repository: https://doi.org/10.17605/OSF.IO/8DS7G.

Research involving human participants, their data, or biological material

Policy information about studies with human participants or human data. See also policy information about sex, gender (identity/presentation), and sexual orientation and race, ethnicity and racism.

Reporting on sex and gender	Sex and gender were not initially considered in the study design. Biological sex was self-reported by participants. Sex demographics of our sample was reported in the methods section. For the initial submission, we did not perform any sex- or gender-based analyses.
Reporting on race, ethnicity, or other socially relevant groupings	NA
Population characteristics	Age (SD): 31 (7.8). Range: 20-45; BMI (SD): 29.2 (7.9) kg/m2. Range: 20-45
Recruitment	Forty-three healthy, native-English-speaking volunteers from the greater Washington, DC metropolitan area were included in this study. Participants were excluded from taking part in the study if they had diabetes, or if they reported any recent weight changes (> 5kg) in the past 6 months, any allergies to food or local anesthetics, any involvement in regular vigorous exercise regimens, daily use of alcohol or illicit drugs in the previous 6 months, or any strict dietary concerns (vegetarian or kosher diet). Participants were also excluded if they had any history of neurological injury, known genetic or medical disorders that may impact the results of cognitive testing and/or neuroimaging, prenatal drug exposure, severely premature birth or birth trauma, past or present psychiatric conditions (e.g., depression or anxiety disorders), current usage of psychotropic medications, or any exclusion criteria for MRI.
Ethics oversight	The institutional review board of the National Institutes of Health approved all procedures and written informed consent was obtained for all subjects.

Note that full information on the approval of the study protocol must also be provided in the manuscript.

Field-specific reporting

Please select the one below that is the best fit for your research. If you are not sure, read the appropriate sections before making your selection.

☒ Life sciences ☐ Behavioural & social sciences ☐ Ecological, evolutionary & environmental sciences

For a reference copy of the document with all sections, see nature.com/documents/nr-reporting-summary-flat.pdf

Life sciences study design

All studies must disclose on these points even when the disclosure is negative.

Sample size	No sample-size calculation was performed for this study. Though the sample size used, 43, is sufficient for within-subjects fMRI design.
Data exclusions	No data were excluded from these analyses.
Replication	At this stage, no efforts were taken to verify the reproducibility of findings.
Randomization	This is not relevant to our study as there were no separate experimental groups.
Blinding	This is not relevant to our study as there were no separate experimental groups.

Reporting for specific materials, systems and methods

We require information from authors about some types of materials, experimental systems and methods used in many studies. Here, indicate whether each material, system or method listed is relevant to your study. If you are not sure if a list item applies to your research, read the appropriate section before selecting a response.

Materials & experimental systems

n/a	Involved in the study
☒	☐ Antibodies
☒	☐ Eukaryotic cell lines
☒	☐ Palaeontology and archaeology
☒	☐ Animals and other organisms
☒	☐ Clinical data
☒	☐ Dual use research of concern
☒	☐ Plants

Methods

n/a	Involved in the study
☒	☐ ChIP-seq
☒	☐ Flow cytometry
☐	☒ MRI-based neuroimaging

Plants

Seed stocks: NA

Novel plant genotypes: NA

Authentication: NA

Magnetic resonance imaging

Experimental design

Design type: Event-related fMRI design

Design specifications: Food Image task: Five exemplar images of each food and non-food object were presented in this task, for a total of 180 food and 45 non-food pictures presented. All pictures were presented for 2.5s in a pseudo-random order optimized for fMRI task design using optseq2 (http://surfer.nmr.mgh.harvard.edu/optseq). Images were followed by variable duration interstimulus intervals (ISI; 2.5 – 12.5s), during which a black fixation cross appeared against a grey background. Subjects were instructed to press a button when two consecutive pictures contained objects with the same name (for example, two consecutive donut images). Food Pleasantness and Self-Control (PSC) Task: In total, subjects viewed 3 different exemplars of 48 types of food. These foods ranged from highly palatable, high calorie foods with high fat and/or sugar content (e.g., cheeseburgers, french fries, pizza, cake, cinnamon rolls, ice cream, etc.) to uncooked fruits and vegetables (grapes, strawberries, cauliflower, broccoli, carrots, etc.). Food pictures were presented for 5s each, separated by the presentation of fixation cross during a variable duration interstimulus interval (mean ISI = 3.7 s; duration 2.5–7.5 s).

Behavioral performance measures: Food Image task: Subjects were instructed to press a button when two consecutive pictures contained objects with the same name (for example, two consecutive donut images).
PSC task: In one task condition, subjects were asked to provide ratings of how pleasant it would be to eat the depicted food at the present moment (Pleasantness condition). Subjects made responses during rating periods using an MR-compatible hand-held scroll wheel, on a number-line which ranged from 1 "Neutral" to 7 "Extremely Pleasant". Subjects rated foods that would be unpleasant to eat with an "X", which was located at the bottom of the number line. In another task condition, subjects rated how much self-control it would take to NOT eat the depicted food at the present moment (Self-Control condition). Subjects rated the amount of self-control required on a number-line which ranged from 1 "Little Self-Control" to 7 "Extreme Self-Control".

Acquisition

Imaging type(s): Functional and Structural

Field strength: 3T

Sequence & imaging parameters: Gradient echo EPI scan: 44 2.8-mm axial slices (orientation: PA, echo time (TE) = 27 ms, repetition time (TR) = 2500 ms, flip angle = 90 degrees, voxel size = 3.4375 × 3.4375 × 2.8 mm). Food Pictures task: 139 TRs; PSC task: 206 TRs.

Area of acquisition: Whole-brain

Diffusion MRI: ☐ Used ☒ Not used

Preprocessing

Preprocessing software	All image pre-processing was performed using AFNI (http://afni.nimh.nih.gov/afni). Pre-processing steps are consistent with previous studies using these task paradigms (Simmons et al., 2014, 2016, 2018; Avery et al., 2018). The first 4 volumes of each EPI time-course (first 3 volumes for PSC task) were excluded from data analysis to allow the fMRI signal to reach longitudinal equilibrium, and a slice timing correction was then applied to the remaining volumes of each EPI scan. A de-spiking interpolation algorithm (AFNI's 3dDespike) was also used to remove transient signal spikes from the EPI data. All EPI volumes were then registered to a base EPI volume using a 6-parameter (3 translations, 3 rotations) motion correction algorithm, and the motion estimates were saved for use as regressors in the subsequent statistical analyses. Volume registration and spatial normalization to Talairach space were implemented in the same transformation step, in order to minimize the number of interpolation steps performed on EPI data. Final voxel resolution was 2x2x2mm3. Following this, smoothing with a 6mm full width at half maximum Gaussian kernel was performed, and the signal intensity for each EPI volume was normalized to reflect percent signal change from each voxel's mean intensity across the time-course.
Normalization	Linear transformation used to normalize anatomical and epi scans to Talairach atlas.
Normalization template	Original Talairach template
Noise and artifact removal	Subject-level regression models also included regressors of non-interest to account for each run's mean, linear, quadratic, and cubic signal trends, as well as the 6 normalized motion parameters (3 translations, 3 rotations) computed during the volume registration preprocessing.
Volume censoring	no volume censoring used

Statistical modeling & inference

Model type and settings	Univariate analyses were whole-brain random effects, paired-sample t-tests. We performed a network clustering approach based on Representational Similarity Analysis. We also performed an RSA using online behavioral data.
Effect(s) tested	We tested the response to food images vs non-food images and the amplitude-modulated response to food images between pleasantness and self-control conditions. For more details, please see Methods section, p. 30-36

Specify type of analysis: ☐ Whole brain ☐ ROI-based ☒ Both

Anatomical location(s)	ROIs were determined by the location of food-responsive clusters within the initial food vs. non-food analysis.
Statistic type for inference (See Eklund et al. 2016)	An initial p-value threshold of $p < 0.001$ was applied to the statistical map. A cluster-size correction of $p < 0.05$ was implemented using AFNI's 3dClustsim, separately within a whole-brain mask and via a small-volume correction applied to a sub-cortical mask.
Correction	With the whole brain and subcortical maps, we used revised versions of AFNI's 3dFWHMx and 3dClustsim to correct for multiple comparisons by generating smoothness and cluster-size estimates using a spherical non-Gaussian spatial autocorrelation function, which has been demonstrated to produce corrected cluster size values approximately equal to those achieved through non-parametric permutation methods (Cox et al., 2017).

Models & analysis

n/a	Involved in the study
☒	☐ Functional and/or effective connectivity
☒	☐ Graph analysis
☒	☐ Multivariate modeling or predictive analysis
